# Resveratrol induces apoptosis of benign prostatic hyperplasia epithelial cell line (BPH-1) through p38 MAPK-FOXO3a pathway

**DOI:** 10.1186/s12906-019-2648-8

**Published:** 2019-08-29

**Authors:** Chao Li, Wan-Li Hu, Meng-Xin Lu, Guan-Fa Xiao

**Affiliations:** 1 Department of Urology, Zhongnan Hospital of Wuhan University, No. 169 DongHu Road, WuChang District, Wuhan, 430000 Hubei People’s Republic of China; 2 Department of Obstetrics and Gynaecology, Wuhan Fourth Hospital; Puai Hospital, Tongji Medical College, Huazhong University of Science and Technology, No.473 HanZheng Street, QiaoKou District, Wuhan, 430000 Hubei People’s Republic of China

**Keywords:** Resveratrol, Benign prostatic hyperplasia, p38 MAPK, FOXO3a, Apoptosis, Reactive oxygen species

## Abstract

**Background:**

Resveratrol is reported to inhibit the growth of prostate, which is characteristic of benign prostatic hyperplasia (BPH) condition. However, the mechanism remains unclear. This study aimed to identify the effects and probable mechanism of resveratrol on BPH.

**Methods:**

We used the BPH epithelial cell line BPH-1 to investigate the effect of resveratrol. Cells were treated with various concentrations of resveratrol, and its effects on cells viability, apoptosis, ROS accumulation, and cell cycle were assessed. Western blot was used to examine activation of p38 MAPK and protein levels of FOXO3a, Bcl2, Bcl-XL, and caspase3. Cells were also co-treated with the p38 MAPK inhibitor SB203580 or ROS scavenger N-Acetyl-L-cysteine (NAC) to further investigate the mechanism.

**Results:**

Resveratrol treatment inhibited the growth of BPH-1 and increased apoptosis of cells. In addition, levels of phosphorylated p38 MAPK level was elevated and FOXO3a repression was observed. Concomitantly, ROS was accumulated. All of these resveratrol-mediated effects were suppressed by additional treatment with SB203580 or NAC. Resveratrol was also found to induce cell cycle arrest at S phase.

**Conclusions:**

Resveratrol can activate p38 MAPK and repress FOXO3a, thereby causing repression of SOD2, catalase, and increase of ROS accumulation, leading to apoptosis in BPH-1 cells.

## Background

Benign prostatic hyperplasia (BPH) is a pathological condition characterized by nonmalignant enlargement of the prostate gland, and is common in elderly men [1, 2]. It is reported that 50% of males show histological characteristics of BPH by the age of 50 years and 80% by the age of 70 years [1]. BPH patients experience frequent micturition or urgent urination, especially at night [3, 4]. Such lower urinary tract symptoms (LUTS) seriously impair the physical and psychological health of BPH patients. Despite several studies, the exact mechanisms and pathogenesis of BPH remains unclear; nonetheless, the pathological progression certainly involves tissue proliferation of epithelia and stroma [5, 6].

Therapy for BPH consists of medical treatment and surgery. Traditional therapy for BPH involves administration of 5α-reductase inhibitors and α blockers, which can shrink the prostate gland or alleviate bladder outlet obstruction (BOO). However, these treatments have many side effects, such as gynecomastia and sexual dysfunction, and is not effective in all patients [7]. Therefore, there is a need for more efficient drugs with less side effects to treat BPH.

MAPK signal transduction pathways have been implicated to play key roles in a number of biological processes, including cell growth, differentiation, apoptosis, inflammation, and responses to environmental stresses [8]. It is reported that MAPK/ERK pathway is essential for the proliferation of human prostate [9, 10]. Resveratrol is a compound found in significant quantities in red wine, grapes, and peanuts [11]. It has anti-proliferation effect in many cancers [12–16] or non-tumor cells [17]. Studies investigating resveratrol have indicated that it can activate p38 MAPK and induce apoptosis of cells [18, 19]. Recently, a report indicated that resveratrol could enhance apoptosis in BPH-induced rats through apoptotic protein regulation, although the exact mechanism of this effect is not elucidated. The current study aimed at exploring the effects of resveratrol on BPH and determining whether MAPK pathway is involved in its effects.

## Methods

### Reagents and antibodies

Resveratrol was purchased from Beyotime Biotechnology (Shanghai, China), while 3-(4,5-dimethylthiazol-2-yl)-2,5-diphenyltetrazolium bromide (MTT), dimethylsulfoxide (DMSO), and dichloro-dihydro-fluorescein diacetate (DCFH-DA) were procured from Sigma. Phosphorylated-p38 MAPK inhibitor SB203580 and ROS scavenger N-Acetyl-L-cysteine (NAC) were provided by MCE. The primary antibodies for p38 MAPK, phosphorylated-p38 MAPK, and Bcl-xl were obtained from Cell Signaling Technology. Antibodies recognizing Foxo3a, Caspase 3, Bcl2, Catalase, and SOD2 were purchased from Proteintech (China), while anti-β actin antibody was purchased from Santa Cruz. The secondary rabbit and mouse-specific antibodies were obtained from Sungene Biotech (China).

### Cell culture and treatment

Benign prostatic hyperplasia epithelial cell line (BPH-1) was obtained from ATCC and cultured in RPMI 1640 medium, supplemented with 10% fetal bovine serum, and placed in warm and moist environment with 5% CO_2_. BPH-1 cells were passaged every 2 days. For treatment, cells were seeded at a density of 30 × 10^4^ per well in 6-well plates, until they reached 80% confluence. They were incubated with various concentrations of resveratrol, resveratrol plus SB203580, or resveratrol plus NAC.

### MTT assay

BPH-1 cells were seeded in 96-well plates (5 × 10^3^ cells per well) and incubated at 37 °C in an incubator containing 5% CO_2_. After adherence, cells were treated with diverse concentrations (0, 10, 20, 30, 40, and 50 μM) of resveratrol and cultured for 2 days. The cell viability was tested each day. Briefly, after administration, the medium was carefully removed and the cells were incubated in 200 μL RPMI 1640 medium containing 0.5 mg/mL MTT at 37 °C for 4 h. Then, the supernatant was discarded, 150 μL DMSO was added to each well, followed by incubation at room temperature for 10 min. Then, light absorbance was measured at 490 nm.

### Apoptosis assessed by flow cytometry

After administration of the indicated drugs, the apoptosis rate was determined by measuring annexin V-FITC and propidium iodide (PI) (Sungene Biotech Annexin V- FITC apoptosis analysis kit, China) via flow cytometry, according to the manufacturer’s instructions.

### Measurement of ROS production

Cells were treated with indicated drugs for 24 h for ROS production measurement, and then harvested, washed with PBS, and incubated with DCFH-DA (10 μM) diluted in 1640 medium without FBS. After incubation for 30 min at 37 °C, the cells were centrifuged and washed with PBS three times to wash away the extracellular DCFH-DA. Then, the cells were submitted to flow cytometry for ROS analysis.

### Cell cycle analysis

For cell cycle analysis, cells were administered the indicated drugs for 24 h, after which they were harvested, centrifuged, washed with PBS, and re-suspended with 1× DNA staining solution containing PI and permeabilization solution (Multi Science cell cycle staining kit, China). After incubation in dark for 30 min, the cells were analyzed by flow cytometry.

### Protein extraction and western blot analysis

RIPA buffer containing protease inhibitor and phosphatase inhibitor were used to lyse the cells for 30 min on ice. Afterwards, cells were centrifuged at 12000 *g* for 15 min to collect the supernatant. Part of the sample was used to measure the protein concentration by BCA assay (Beyotime Biotechnology BCA Protein Assay Kit, China). The remaining sample was boiled at 100 °C with 5× loading buffer for 10 min and used for western blot analysis, which was performed as described previously [20]. The β-actin was used as a loading control.

### Statistical analyses

Each experiment was performed more than three times and data are presented as mean ± standard deviation (SD). One-way ANOVA and student’s *t*-test were used to analyze the data. A *P* value < 0.05 was considered to be statistically significant.

## Results

### Resveratrol inhibited the growth and promoted apoptosis of BPH-1

**Fig. 1:**
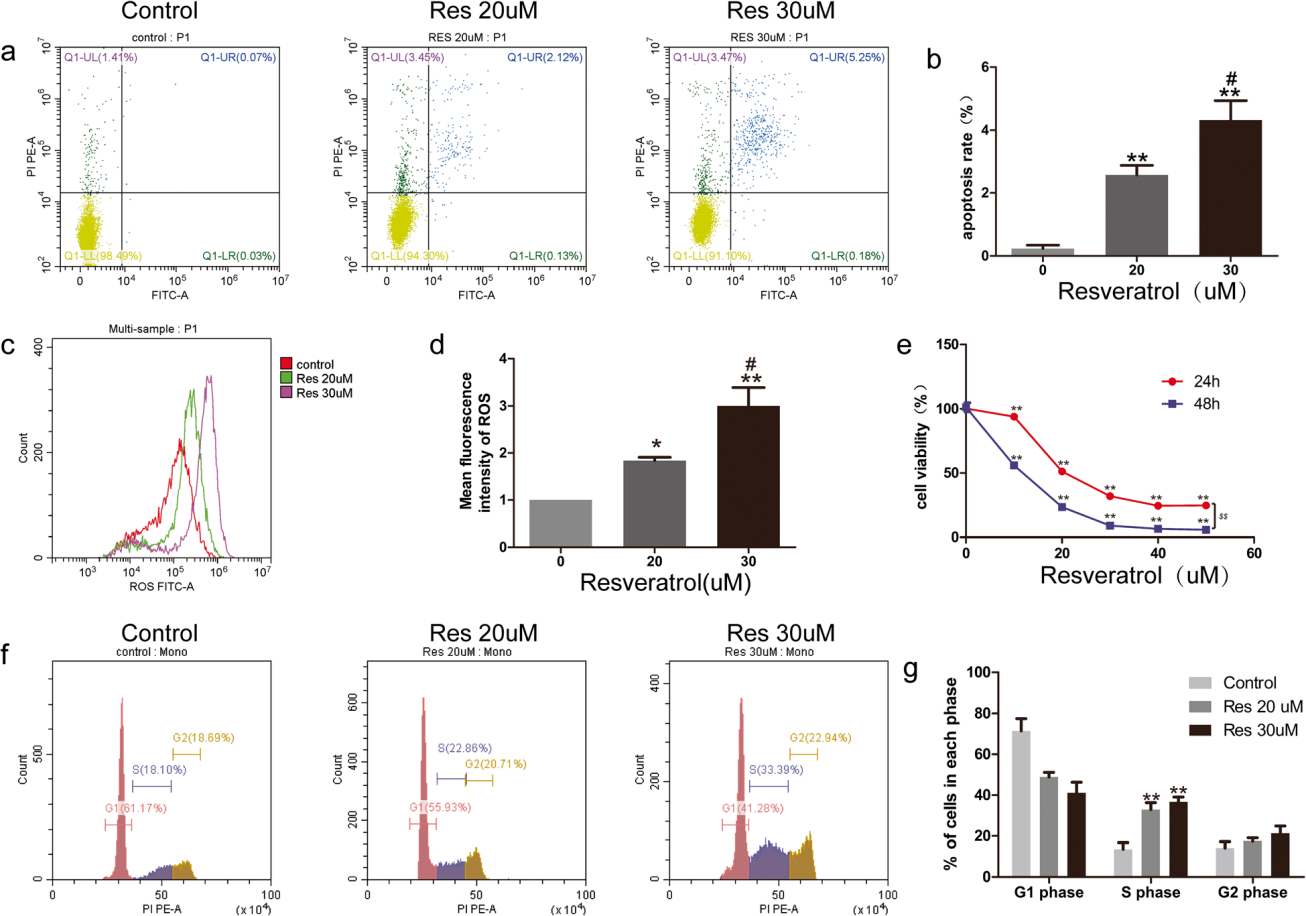
The effects of resveratrol on apoptosis, ROS and cell cycle. **a, b** Flow cytometry analysis of apoptosis. **c, d** Measurement of ROS production by flow cytometry. **e** MTT assay detected on BPH-1 after treatment. **f, g** Cell cycle analysis by flow cytometry. Res represents resveratrol, *P < 0.05, **P < 0.01(compared with vehicle-treated control), #P < 0.05, ##P < 0.01(compared with Res 20 μM treating), $$P < 0.01(compared with 48 h treating)

To investigate the effect of resveratrol on BPH, we used the BPH-1 cell line as our model. The BPH-1 cells were treated with various concentrations of resveratrol for two days, after which the cell viability was markedly reduced in a time and dose-dependent manner (Fig. 1e). Treatment with 20 μM resveratrol for 24 h inhibited almost half of the cells, while more than 90% cells were inhibited after administration of ≥30 μM resveratrol. Furthermore, BPH-1 treatment also induced cell morphological changes; the cell shape was transformed from polygon to spindle, and numerous fragments of cells were observed to be floating on the medium.

To further clarify the growth-inhibitory effect of resveratrol, we investigated apoptosis of BPH-1 using flow cytometry. After treatment with resveratrol for 24 h, the apoptosis rate was significantly enhanced (*P* < 0.01), and increased in a concentration dependent manner (Fig. 1a and b). Together, these data indicated that resveratrol exerts significant cytotoxic effect upon BPH-1 in a dose and time-dependent manner.

### ROS accumulation and cell cycle arrest were observed in resveratrol treated BPH-1 cells

Since apoptosis can be regulated through oxidative stress or cell cycle, we evaluated each of these after administration of resveratrol. Compared with vehicle-treated control, the ROS level was notably elevated by 1.8 fold (*P* < 0.05) and 3 fold (*P* < 0.01) after incubation with 20 μM and 30 μM resveratrol, respectively (Fig. 1c and d). Analysis of cell cycle showed a decreased percentage of cells in G1 phase and high percentage of cells in the S phase after resveratrol treatment (Fig. 1f and g). These results indicated that ROS accumulation and cell cycle arrest might be involved in the effect of resveratrol.

### p38 MAPK activation and FOXO3a down-regulation were involved in the apoptosis of BPH-1

**Fig. 2:**
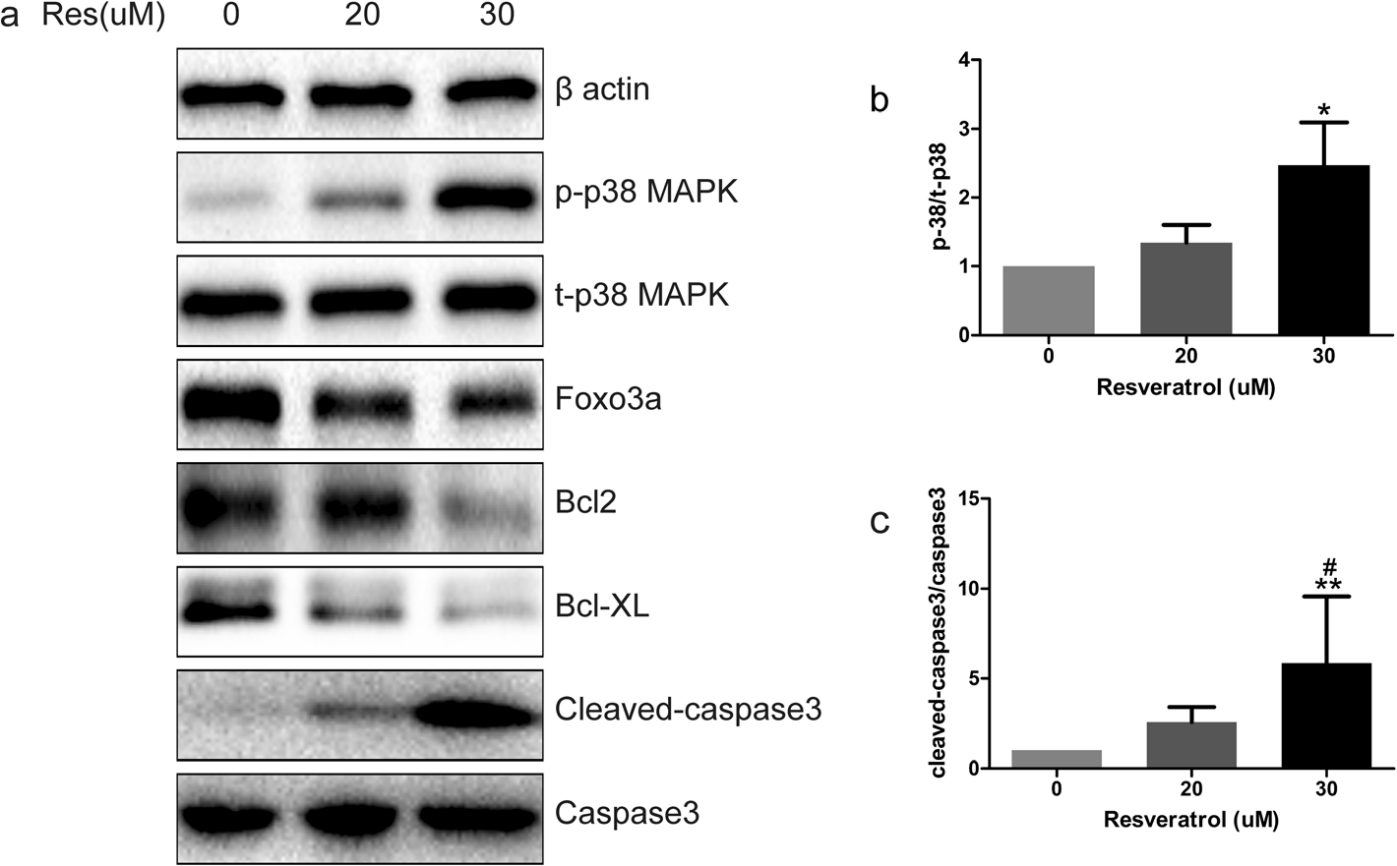
Western blot analysis after administration. **a** Western blot stripes of βactin, p-p38MAPK, p38MAPK, Foxo3a, Bcl2, Bcl-XL, Cleaved-caspase3, Caspase3. **b, c** One way anova analysis was used to analysis the p-38 and cleaved-caspase3 protein expression, *P < 0.05(compared with vehicle-treated control), #P < 0.05(compared with Res 20 μM treating)

The p38 MAPK pathway is thought to be activated in response to various environmental and cellular stresses, inflammation, and other signals [21]. Since our results suggested increased ROS accumulation by resveratrol treatment, we examined whether this involved the p38 MAPK pathway. Western blot analysis indicated that phosphorylated-p38 MAPK level was significantly increased after resveratrol treatment, although the total p38 MAPK level remained unchanged (Fig. 2a and b). In addition, the level of phosphorylated-p38 MAPK increased with increasing concentrations of resveratrol. Furthermore, the treatment down-regulated FOXO3a protein expression (Fig. 2a). Interestingly, the treatment also down-regulated the expression of anti-apoptotic factors Bcl2 and Bcl-XL, while cleaved caspase3 level was significantly enhanced (Fig. 2a and c). These results suggested that resveratrol may induce apoptosis via p38 MAPK activation and FOXO3a repression.

### p38 MAPK inhibitor SB203580 and NAC can alleviate resveratrol-induced apoptosis and ROS accumulation

**Fig. 3:**
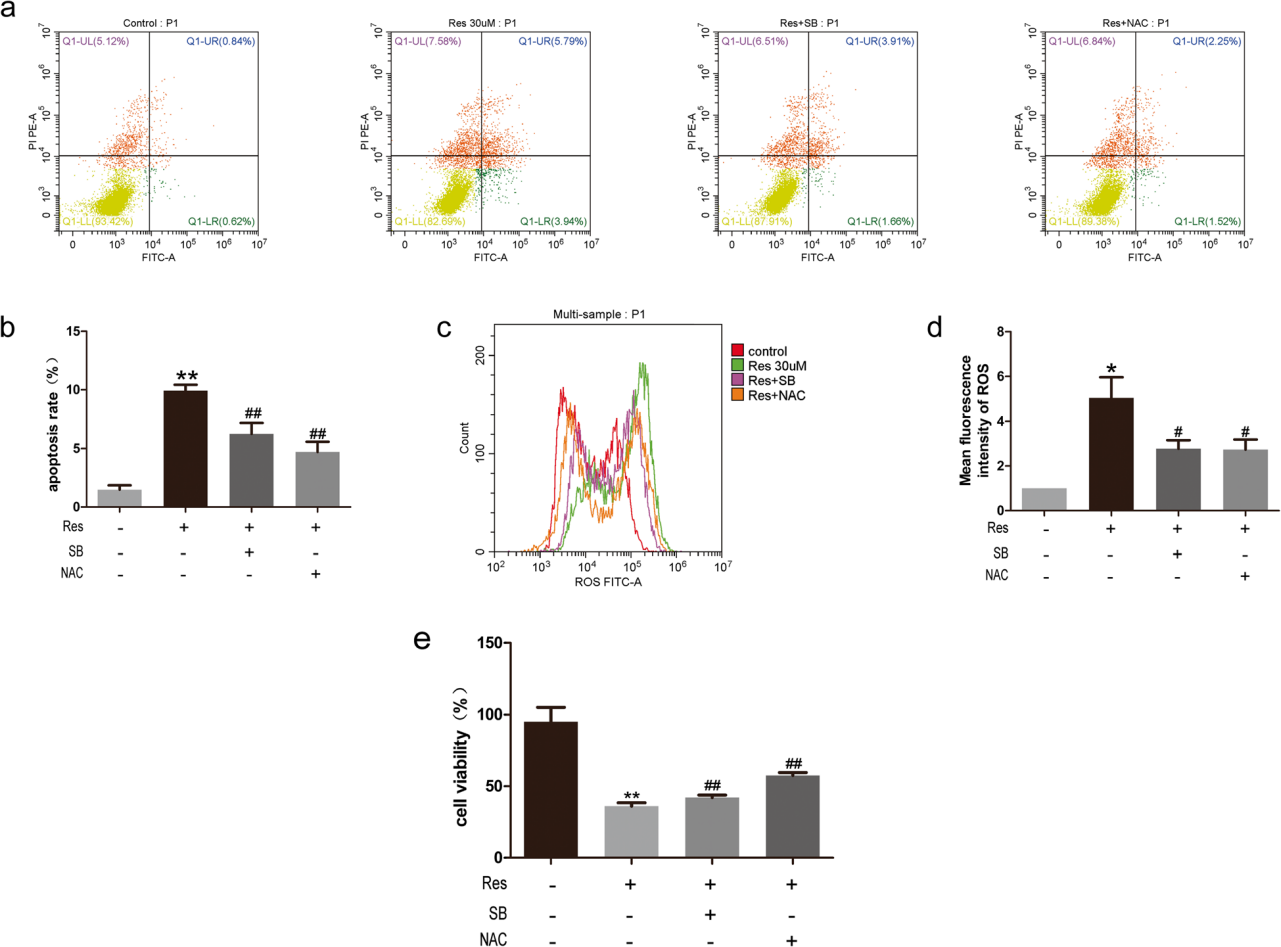
SB203580 or NAC relieved the resveratrol induced growth inhibition, apoptosis and ROS accumulation. **a, b** Flow cytometry analysis of apoptosis. **c, d** Measurement of ROS production by flow cytometry. **e** MTT assay after treatment. SB and NAC are the abbreviation of SB203580 and N-Acetyl-L-cysteine, BPH-1 was treated with 30 μM Res, 30 μM Res plus 5 μM SB and 30 μM Res plus 5 mM NAC. student’s *t*-test was used to analysis the data, *P < 0.05,**P < 0.01 (compared with vehicle-treated control), #P < 0.05, ##P < 0.01 (compared with Res 30 μM treating)

Next, cells were treated with the p38 MAPK inhibitor SB203580 to investigate whether inhibition of p38 MAPK pathway could reduce apoptosis of BPH-1. The cell viability was significantly increased upon administration of resveratrol plus SB203580 (*p* < 0.01) (Fig. 3e). Moreover, resveratrol-induced apoptosis and ROS accumulation were also ameliorated upon additional treatment of SB203580 (Fig. 3a–d). Treatment with the ROS scavenger NAC elicited similar effects (Fig. 3a–e). Together, these data revealed that the apoptotic effect of resveratrol was mediated via the p38 MAPK pathway and ROS participated in the activation of p38 MAPK.

### Changes in protein level after treatment of BPH-1 with SB203580 and NAC

**Fig. 4:**
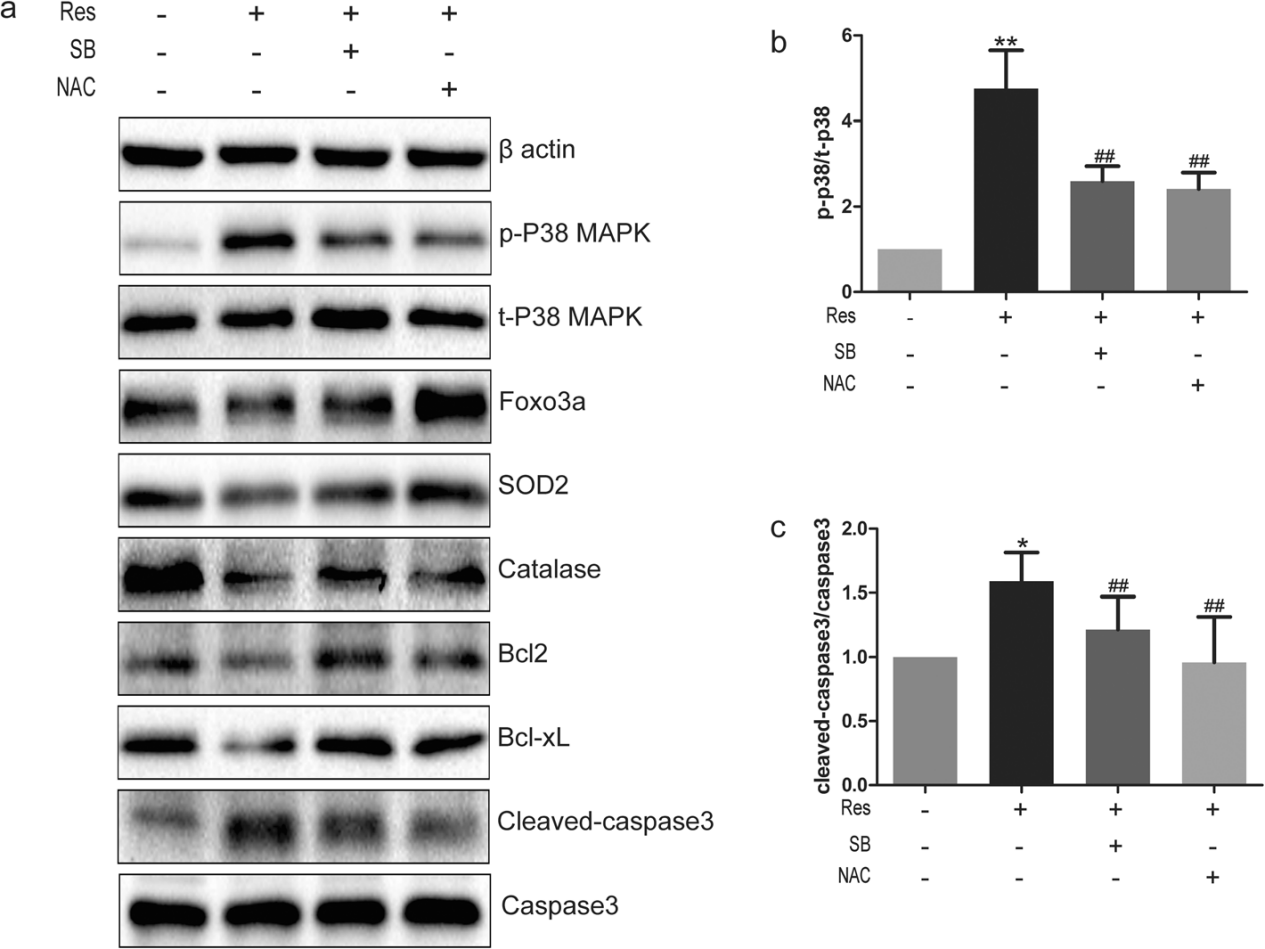
Western blot analysis after co-incubating with SB203580 or NAC**. a** Western blot stripes of β actin, p-p38MAPK, p38MAPK, SOD2, Catalase, Foxo3a, Bcl2, Bcl-XL, Cleaved-caspase3, Caspase3. **b, c** Relative expression of p-38 or cleaved-caspase3 were analysed by student’s *t*-test. *P < 0.05,**P < 0.01 (compared with vehicle-treated control), ##P < 0.01 (compared with Res 30 μM treating)

Protein analysis indicated that treatment with SB203580 and NAC significantly repressed the phosphorylation of p38 MAPK (Fig. 4a and b), and relieved the suppression of FOXO3a protein expression (Fig. 4a). Compared with the resveratrol treatment alone, co-incubation with SB203580 or NAC improved the expression of SOD2 and catalase. Furthermore, Bcl2 and Bcl-XL protein levels were elevated (Fig. 4a), while level of cleaved caspase3 was decreased (Fig. 4a and c) upon the additional treatments. These results indicated that resveratrol may activate p38 MAPK and repress the expression of FOXO3a, causing less ROS clearance and subsequent triggering of apoptosis.

## Discussion

The most important characteristic of BPH is increased cell proliferation and reduced apoptosis [22]. Therefore, a therapeutic strategy that can enhance apoptosis or cell death in BPH tissue is desirable. Previous study suggested that resveratrol decreases prostate weight in BPH rats by regulating the expression levels of apoptosis proteins [23]; however, the exact mechanism was not elucidated. In the current study, we demonstrated that resveratrol induces apoptosis via p38 MAPK activation, ROS accumulation, and FOXO3a repression.

MAPKs are involved in four major pathways: the extracellular-signal regulated kinase (ERK), c-jun N-terminal kinase (JNK), Big MAP kinase (BMK), and p38 MAPK [24]. Among these, the p38 MAPK is activated in response to cellular stress such as oxidative stress, UV, DNA damage, and inflammatory cytokines [25]. In our study, resveratrol treatment resulted in enhanced activation of p38 MAPK. Notably, treatment with p38 MAPK inhibitor significantly reduced resveratrol-induced phosphorylation of p38 MAPK, ROS accumulation and apoptosis rate. Additionally, treatment with NAC, a ROS scavenger, also produced similar effects. These observations indicated that the apoptotic effect of resveratrol might be exerted via p38 MAPK activation and ROS accumulation. Furthermore, there seems to be a link between p38 MAPK activation and ROS, since NAC treatment could reduce phosphorylation of p38 MAPK.

Studies have reported that FOXO3a, a transcriptional regulator, can trigger apoptosis via up-regulation of pro-apoptotic and down-regulation of anti-apoptotic genes [26, 27]. SOD2 and catalase are also vital anti-oxidant enzymes and transcriptional targets of FOXO3a [28]. Our study showed a decreased expression of FOXO3a protein, and concomitant low expression of SOD2 and catalase upon resveratrol treatment. However, this effect could be suppressed by additional treatments with SB203580 or NAC. This indicated that resveratrol-mediated p38 MAPK activation might repress the expression of FOXO3a, and thereby reduce levels of SOD2 and catalase, enhance pro-apoptosis gene expression, and repress anti-apoptosis genes expression, leading to apoptosis in BPH-1 cells.

However, our study revealed that mRNA expression of FOXO3a (data not shown) was not altered after resveratrol treatment, contrary to its protein expression. This suggested that post-translational modifications might be involved in FOXO3a repression. In addition, our observations revealed that resveratrol induces cell cycle arrest at S phase, although the exact mechanism is still not clarified. We aslo observed the different results from those which indicate that resveratrol is a free radical scavenger and an antioxidant [29, 30], in our view, the distinct observation may be due to the cell types, concentrations, time and the diverse conditions before treating with resveratrol (high glucose, hydrogen peroxide) [31]. To the best of our knowledge, this is the first study to reval potential mechanism of the apoptosis effect of resveratrol on BPH-1. It has already been reported that resveratrol can regulate androgen receptors or estrogen receptors in distinct ways [13, 32–34]. These observations, combined with our findings make resveratrol a potential therapeutic agent to treat BPH.

## Conclusions

In conclusion, our study shows that resveratrol can induce apoptosis and ROS accumulation in prostatic hyperplasia epithelial cell line BPH-1 via p38 MAPK regulated FOXO3a repression.

## Data Availability

The datasets generated and analyzed during the current study are available through the corresponding author on reasonable request.
